# Exposure to Engineered Nanomaterials: Impact on DNA Repair Pathways

**DOI:** 10.3390/ijms18071515

**Published:** 2017-07-13

**Authors:** Neenu Singh, Bryant C. Nelson, Leona D. Scanlan, Erdem Coskun, Pawel Jaruga, Shareen H. Doak

**Affiliations:** 1 School of Allied Health Sciences, Faculty of Health & Life Sciences, De Montfort University, The Gateway, Leicester LE1 9BH, UK; 2 Material Measurement Laboratory, Biosystems and Biomaterials Division, National Institute of Standards and Technology, 100 Bureau Drive, Gaithersburg, MD 20899, USA; bryant.nelson@nist.gov; 3 Material Measurement Laboratory, Biomolecular Measurement Division, National Institute of Standards and Technology, 100 Bureau Drive, Gaithersburg, MD 20899, USA; scanlan.leona@gmail.com (L.D.S.); erdem.coskun@nist.gov (E.C.); pawel.jaruga@nist.gov (P.J.); 4 Swansea University Medical School, Institute of Life Science, Centre for NanoHealth, Swansea University Medical School, Wales, SA2 8PP, UK

**Keywords:** engineered nanomaterials, DNA damage, nanotoxicity, DNA repair proteins/genes, DNA repair pathways

## Abstract

Some engineered nanomaterials (ENMs) may have the potential to cause damage to the genetic material in living systems. The mechanistic machinery functioning at the cellular/molecular level, in the form of DNA repair processes, has evolved to help circumvent DNA damage caused by exposure to a variety of foreign substances. Recent studies have contributed to our understanding of the various DNA damage repair pathways involved in the processing of DNA damage. However, the vast array of ENMs may present a relatively new challenge to the integrity of the human genome; therefore, the potential hazard posed by some ENMs necessitates the evaluation and understanding of ENM-induced DNA damage repair pathways. This review focuses on recent studies highlighting the differential regulation of DNA repair pathways, in response to a variety of ENMs, and discusses the various factors that dictate aberrant repair processes, including intracellular signalling, spatial interactions and ENM-specific responses.

## 1. Introduction

The unique properties of engineered nanomaterials (ENMs), intentionally manufactured particles or objects with at least one dimension in the size range of 1 nm to 100 nm (with 50% or more particles in the number size distribution), have contributed to the exponential growth and innovative advances in nanotechnology [1,2,3]. Monodisperse ENMs, as well as agglomerated and/or aggregated ENMs display a diverse range of magnetic, optical, electrical, catalytic and antibacterial properties which has led to their incorporation into a plethora of consumer and industrial products. Some of these products include personal care items (cosmetics, sun-creams, scratch resistant nail polishes, deodorants, toothpastes, etc.) medical devices (tissue scaffolds, drug delivery systems, orthopaedic implants, imaging modalities, biosensors, etc.) and nanoelectronics (field effect transistors, photonic crystals, field emission displays, etc.). The most commonly used ENMs include metal nanoparticles—NPs (silver, gold, cobalt, cobalt-chromium), metal oxide NPs (titanium dioxide, zinc oxide, silica, iron oxide), quantum dots (cadmium, tellurium, selenium) and carbon nanomaterials [3].

**Figure 1:**
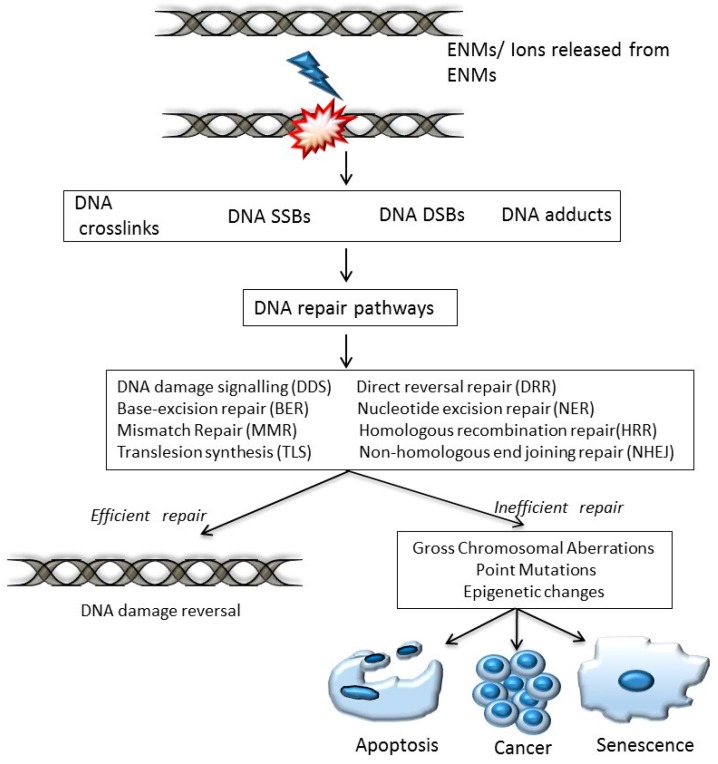
Schematic to illustrate various types of DNA damage caused by engineered nanomaterials (ENMs) that may result in efficient or inefficient repair activity, leading to either DNA damage reversal or progression to carcinogenesis, apoptosis and/or senescence, respectively. SSB: single strand breaks, DSB: double strand breaks.

Ongoing research on evaluating the safety of ENMs has highlighted the potential of these nano-entities to cause perturbation of various cellular pathways and functional processes [2,3]. The alterations that may occur in the intracellular milieu in response to ENM-exposure can have unpredictable consequences on the functioning of the entire cellular system, as well as on the fidelity of DNA replication and cell division [4]. Cellular exposure to some ENMs has been linked to DNA damage resulting in wide ranging DNA lesions, which include genome rearrangements, single strand breaks (SSBs), double strand breaks (DSBs), intra/inter strand breaks (SBs) and the formation of modified bases (thymine glycol, 5-hydroxy-5-methylhydantoin, 8-hydroxyguanine) [5,6]. These different types of DNA lesions can lead to chromosomal aberrations, gene mutations, apoptosis, carcinogenesis or cellular senescence if left unrepaired [7] (Figure 1). Therefore, thorough studies on DNA damage response and repair related genes, pertaining to ENM exposure testing, are necessary for evaluating and characterizing the safety of ENMs.

The integrity of the genome is maintained at three levels. Level 1 involves phase I (involved in hydrolysis, oxidation etc.) and/or phase II (involved in methylation, conjugation with glutathione etc.) metabolizing enzymes that can process, inactivate or intercept the mechanistic processes that lead to ENM-induced DNA damage [8]. For example, a main antioxidant defence molecule, glutathione (along with antioxidant enzymes) binds and neutralizes reactive oxygen species (ROS) [9]. Although these defences at level 1 are generally effective, the generation of excess ROS subsequent to ENM exposure can tip the balance in favour of oxidative stress, which can lead to mutations and chromosomal aberrations [10,11]. Level 2 includes signal transduction pathways involving molecules that act as sensors for DNA damage and activate defence checkpoints. Level 3 mainly involves DNA repair processes, which play a pivotal role in maintaining the integrity of the genome by repairing DNA damage [8].

DNA repair is a complex process: it is comprised of >168 genes (that encode for proteins) involved in numerous, diverse processes encompassing intracellular signalling, cell cycle checkpoints, enzymatic reactions and chemical and structural modifications and transformations which eventually culminate in DNA repair [12]. Each pathway is represented by a set of proteins and enzymes with distinct functions and enzymatic activities. Many of the proteins implicated in DNA repair are well-defined in terms of kinetic activity, substrate specificity, mode of action and 3D structure. Therefore, knowledge about the DNA repair systems and their components is critical to our understanding of how cells control and repair the constantly occurring damage in their genomes.

There are multiple DNA repair pathways targeting various levels and extent of DNA damage. Researchers over the last few decades have progressively deciphered and revealed responses to DNA damage (including ENM-induced); currently, the DNA damage repair pathways can be divided into eight major categories:

- DNA damage signalling (DDS): this pathway is induced in response to DNA damage caused by various agents including environmental, ENM and endogenous. DDS pathways are programmed to induce several cellular responses including checkpoint activity, triggering of apoptotic pathways and DNA repair [13].
- Direct reversal repair (DRR): reverses/eliminates the DNA damage caused by chemical reversal or modification by restoring the original nucleotide. It is also known as direct DNA damage reversal.
- Base-excision repair (BER): this repair mechanism is initiated by the excision of modified bases from DNA by DNA glycosylases. The length of the DNA that needs to undergo re-synthesis can be variable; thus, the pathway can be subdivided into short-path or long-path BER. Although various pathways are involved in this repair process, one of the most widely studied mechanisms that triggers the BER pathway is oxidatively induced damage. Since oxidative stress is one of the most common mechanisms of ENM-induced DNA damage, oxidatively induced DNA lesions are predominantly repaired by the BER pathway (see Table 1). The key enzymes involved in the BER process are DNA glycosylases, which remove damaged bases by cleavage of the N-glycosylic bonds (between the bases and deoxyribose moieties) of the nucleotide residues. The DNA glycosylase action is followed by an incision step, DNA synthesis, an excision step, and DNA ligation. Various metal oxide based ENMs, quantum dots and carbon nanomaterials have been implicated in activating the BER pathway (Table 1).
- Nucleotide excision repair (NER): is involved in removing bulky DNA adducts. The damage from the active strand of transcribed DNA and DNA damage elsewhere in the genome is removed in this pathway by transcription-coupled repair and global genome repair, respectively. Silver and cadmium based ENMs have been shown to interfere with the NER pathway (Table 1).
- Mismatch repair (MMR): this pathway is involved in post-replicational DNA repair that removes errors including mismatched nucleotides, insertions, deletions, etc.
- Homologous recombination repair (HRR): this pathway involves repair of DSBs using the homologous DNA strand as a template for re-synthesis.
- Non-homologous end joining repair (NHEJ): helps to ligate the DNA ends resulting from DSBs.
- Translesion synthesis (TLS): this pathway employs specialized polymerases that use damaged DNA as templates, to finish replication across lesions. Although the mechanism is error-prone, and cell survival may be associated with an increased risk of mutagenesis/carcinogenesis, it helps to prevent a stalled replication fork.

## 2. Activation/Up-Regulation of DNA Damage Signalling Pathways

DNA repair pathways/proteins seldom work in isolation in the cell, i.e., the repair pathways are interdependent and interconnected via shared proteins and components of the DNA repair system. More than one pathway may be up-regulated/down-regulated in response to cellular exposure by a given ENM. Moreover, repair pathways (genes/proteins/enzymes) induced because of DNA damage do not follow a similar trend with respect to being up-regulated or down-regulated; different studies on ENM exposure have shown varied DNA damage responses, i.e., the same gene/protein is up-regulated in one study, while being down-regulated in another ENM-exposure study. For example, several studies have shown apurinic/apyrimidinic endonuclease (APEX), involved in the BER pathway to be either up-regulated or down-regulated in response to exposure by ENMs (Table 2). This lack of a trend may be due to differences in physico-chemical characteristics (e.g., composition, size, structure, charge, morphology, coating, presence of impurities due to synthesis processes) in the tested ENMs or to differences in exposure concentrations, cell lines utilized and other experimental factors.

## 3. Up-Regulation of DNA Repair Genes

Prasad et al. recently showed that titanium dioxide NPs (TiO_2_ NPs) induce the activation of serine/threonine kinase ATM/Chk2, involved in the DDS signalling pathway [13] (see Table 1). The study showed that TiO_2_ NPs behave like ionizing radiation (IR), a well-known trigger for both the ATM/Chk2 pathway and the intra-S-phase DNA-damage checkpoint response [13,26]. TiO_2_ NPs-induced increased expression (>1.5-fold) of ATM in hepatocellular carcinoma cells (HepG2), which is consistent with the induction of DSBs, chromatin condensation, nuclear fragmentation and apoptosis due to increased ROS production and subsequent DNA damage [23]. This enhanced ROS generation, which correlated with toll-like receptor 4 (TLR4) over-expression (vs. TLR3 over-expression, which protects against ROS-induced DNA damage) activated caspase-3 and oxidative stress-induced apoptosis. Similar increases in the expression of ATM (but decreased expression of ATR) with a corresponding increase in DNA damage (as indicated by micronucleus frequency), are attributed to increased ROS generation and oxidative stress; the experimental evidence suggested that DSBs were involved [27]. Therefore, up-regulation of the ATM protein is associated with its role as a DNA damage sensor that activates checkpoint signalling events subsequent to DSBs induced by TiO_2_ NPs.

Apart from DDS, various other pathways have been shown to be activated by some ENMs. Tang et al., have demonstrated similar up-regulation of certain DNA repair genes in zebrafish liver cells including *Ku80* (NHEJ), *OGG1* (BER), *XPC* (NER) and *XPA* (NER) using cytotoxic concentrations of CdSO_4_ salt or CdTe quantum dots (QDs) [19]. A QD exposure study by the same group tested cadmium selenide/zinc sulfide (CdTe/ZnS) core-shell QDs on the fresh water crustacean *Daphnia pulex*, and showed significant increases in OGG1 levels in response to CdTe QDs, but not for the CdTe/ZnS QD exposure. Genes involved in the NER pathway, namely *XPA* and *XPC*, showed significant up-regulation in response to treatment with both CdTe and CdSe/ZnS QDs. In addition to the BER and NER pathways, the MMR pathway was also affected in response to CdTe and CdSe/ZnS QDs [20]. Therefore, subsequent to exposure by a particular ENM, various genes of the repair pathways can be triggered, which work in conjunction with other proteins/enzymes/co-factors to eliminate the DNA damage. For example, copper oxide (CuO) NPs were shown to upregulate the expression of two DNA damage repair proteins RAD51 (HRR) and MSH2 (MMR) in lung epithelial cells, while up-regulation of *RAD51* along with increased levels of *Ku70* (implicated in NHEJ pathway) was observed in in THP-1 cells exposed to photocopier-emitted NPs [21,22].

Interestingly, some studies have also shown tissue-specific up-regulation of DNA damage genes/proteins in response to ENM exposure. Van Berlo et al. observed increased levels of DNA damage response genes *OGG1* and *APE1* in C57BL/6J mice exposed to carbon NPs, in a short-term inhalation study [18]. However, elevated mRNA levels of the two genes were seen in lung tissue, while the olfactory bulb cerebellum and other parts of the mice brain were not affected. Nevertheless, long-term studies are needed to evaluate any adverse effects on the brain, particularly with respect to other and perhaps more toxic ENMs, which may be released into the environment.

Similarly, a tissue-specific response was seen in a TiO_2_ NP exposure study that used three model cell lines representing an alveolar-capillary barrier. The cell system consisted of alveolar macrophage-like THP-1 cells, alveolar epithelial A549 cells and human pulmonary microvascular endothelial, HPMEC-ST1.6R, cells [24]. Following exposure to the test ENM, significant levels of ROS were generated in all three cell lines. Differentiated THP-1 macrophages showed increased phosphorylation of ATR and ATM with increasing concentrations of TiO_2_-NPs, (200 to 800 μg/mL). This correlated with increased phosphorylation of H2AX histone (γH2AX) revealing a link between deleterious DNA lesions and activation of the DNA damage repair pathway [24]. On the other hand, in HPMEC-ST1.6R cells, phosphorylation of H2AX histones did not correlate with activation of ATR or ATM proteins. However, an increased phosphorylation of p53 and checkpoint protein CHK1A was observed to correlate with cell cycle arrest. Interestingly, the A549 cell line showed no activation of signalling pathways related to DNA damage. This study thus sheds light on the differential profile of tissue specific DNA repair responses generated by the three cell lines under investigation, with only THP-1 and HPMEC-ST1.6R cells showing apoptosis, sensitivity to redox changes and concomitant activation of DNA damage and repair proteins.

## 4. Inactivation/Downregulation of DNA Repair Pathway Genes

Any causative agent that results in DNA damage may be anticipated to bear a positive correlation to an increased repair capacity, as described in studies in the previous section. This is possible by induction of DNA damage pathway genes that ensure high fidelity DNA synthesis to rectify the observed damage in order to maintain genome stability. However, a number of genes that participate in DNA damage repair processes and induction of cell cycle checkpoints are either up-regulated (e.g., *RAD9*, *PARP1*) or down-regulated/mutated (e.g., *BRCA1/2*, *ATM* and *TP53*); diminished repair capacity has also been associated with carcinogenesis [7]. Many ENM exposure studies have shown downregulation in the expression/activity of key candidate genes/proteins/enzymes involved in DNA repair pathways (Table 2).

Such downregulation of genes involved in the BER pathway has been shown by Kovvuru et al. following exposure to polyvinylpyrrolidone (PVP) coated AgNPs; the genes involved mediate and contribute to the observed oxidatively induced DNA damage, DSBs, and chromosomal damage in peripheral blood and bone marrow [15]. The study provided evidence that some of the BER pathway genes, which play a pivotal role in the repair of oxidatively induced DNA damage, were down-regulated—these genes include *NEIL1*, *NEIL3*, *NTHL1*, *MUTHY*, *APEX2*, *RPA1*, *XRCC1*, *PARP1* and *LIG1* (Table 2). The genes and proteins implicated in the BER pathway, are collectively involved in (1) recognition and base excision of ENM-induced DNA damage (2) repair, intermediate processing, synthesis and (3) nick sealing or ligation.

A correlation between chromosomal damage and impairment of repair pathways was also established using mice deficient in a BER pathway protein MutY homologue (MUTYH). These animal models were observed to be hypersensitive to PVP-coated AgNPs and resulted in increased micronuclei frequency indicating chromosomal damage [15]. MUTYH knock-down is also associated with decreased ATR, CHK1 and CHK2 phosphorylation induced by hydroxyurea, ultraviolet light and topoisomerase II inhibitor treatment [28,29]. This downregulation correlated with decreased apoptosis and reduced activation of ATR, which regulates cell cycle arrest and apoptosis [29]. MUTYH has been described as a trigger for cell death pathways in cells that have accumulated DNA lesions and SSBs [30], thus protecting the cells from permanent genome alterations in the form of damaged DNA. The study on AgNPs sheds light on the importance of DNA damage checkpoint activation as any defects in the execution of apoptosis may impact genomic integrity; it also highlights the interplay of different repair pathways that work in co-ordination to maintain stability in the genome. Other than downregulating key genes related to the BER pathway, genes implicated in other pathways were also down-regulated or up-regulated in response to treatment by PVP-coated AgNPs (Table 1).

The downregulation of DNA repair proteins/genes and its link to ROS induced DNA damage has been studied in depth by Pati et al. [25]. The authors observed a decreased expression of two DNA repair proteins that play an important role in the BER pathway, POLB (DNA polymerase β) and FEN1 (Flap endonuclease 1), in zinc oxide (ZnO) NP-treated macrophages. This correlated with ZnO NP-induced micronuclei frequency, chromosomal disintegration, cellular protrusions, cytotoxicity, reduced cell migration, phosphorylation of the H2A histone family and modulation in actin polymerization in peripheral blood macrophages and bone marrow cells. Interestingly, treatment with N-acetyl cysteine (NAC) after ZnO NP exposure alleviated the observed genotoxicity and clastogenicity and up-regulated the expression of DNA repair genes. This finding may indicate that ZnO NPs exhibit genotoxic, clastogenic, cytotoxic and actin depolymerization effects via generation of ROS in macrophages.

In a recent study comparing the responses of normal cells vs. cancer cells following exposure to AgNPs, both inhibition and activation of DNA repair responses were demonstrated. A comparison of normal lung fibroblasts (IMR-90) cells vs. U251 glioblastoma cancer cells revealed increased ATM and ATR levels, which activate downstream targets for DNA repair in response to DSB [14]. The gene expression results were associated with increased γ-H2AX foci in U251 cells as compared to IMR-90 cells, suggesting that the cancer cells were more prone to the accumulation of DSBs in response to AgNP exposure. The authors showed down-regulation of BER pathway genes (*MBD4*, *PCNA*, *APEX1*, *OGG1* and *MUTYH*) and MMR pathway genes (*PMS1* and *MSH2*) in the IMR-90 cell line, indicating differential gene expression in normal vs. cancer cells [14]. Additionally, the study showed downregulation of *BRCA1* and MMR-dependent *ABL1* gene expression in IMR-90 cells in comparison to the increased expression of *BRCA1* and *ABL1* in glioblastoma cells; indicating involvement of the HRR and MMR pathways. However, the molecular mechanisms of these alterations and regulation have yet to be elucidated.

A relatively new method (multiplexed excision/synthesis assay) developed by Millau et al., which evaluates repair capacity by both the NER and BER pathways was utilised to evaluate the repair ability of A549 cells exposed to TiO_2_ NPs [31]. The TiO_2_ NPs inactivated both the NER and BER pathways, thus impairing the cells’ ability to repair damage to DNA. The induction of DNA damage coupled with the compromised repair ability suggest a synergistic response to TiO_2_ NPs exposure that could potentially lead to mutagenesis or carcinogenesis. However, further detailed studies are necessary to corroborate the results.

Although inefficient/down-regulated repair systems could have unfavourable consequences on various cellular functions, unrepaired DNA damage can have positive implications in cancer therapy. An interesting study based on hybrid NPs composed of bioactive quinacrine (Q, an ideal antimalarial agent) and AgNPs (QAgNPs) demonstrated the capacity to inhibit the BER pathway and subsequently induce apoptosis in cancer cells, thus offering potential new directions in anticancer treatment for oral squamous cell carcinoma [17]. The inherent high DNA repair efficiency in cancer stem cells (CSCs) poses a challenge for chemotherapeutic drug treatment, as CSCs avoid DNA damage-mediated apoptosis during cancer therapy. However, treatment with QAgNPs showed significant reduction of LIG1, FEN1 and POLB, POLD1 (DNA polymerase δ) and POLE (DNA polymerases ε), showing the involvement of the BER pathway, while PRKDC (protein kinase, DNA-activated, catalytic polypeptide) and RAD51, components of NHEJ and HRR respectively, remained unaltered. These results corresponded to a >5-fold increase in the expression of γ-H2AX, indicating sustained DNA damage and formation of DSBs, which is associated with compromised BER activity within the cells and resultant apoptosis. Interestingly, no significant changes were observed when the cells were treated with either quinacrine (NQC) or AgNPs alone.

Steric hindrance is another important mechanism that governs the decreases in DNA repair protein activity. An interesting study on carbon ENMs (cENMs) revealed that these ENMs could physically perturb the incision activity of APEX1 [32]. The study showed that the cENMs were susceptible to adsorption onto custom synthesized DNA oligonucleotides with abasic sites. This study in a cell-free system effectively demonstrated that APE1’s accessibility to the DNA lesions (abasic sites) could be sterically hindered, thereby inhibiting its enzymatic incision activity. This conformational alteration, resulting in stalled repair activity, could potentially lead to mutagenesis through impaired DNA repair processes in a cellular environment.

Release of ions governs repair capacity: co-exposure studies using Cd^2+^ or CdTe QDs and benzo[*a*]pyrene-7,8-diol-9,10-epoxide, (BPDE—a toxic compound that interacts covalently with DNA base pairs and forms adducts, thus leading to DNA damage and carcinogenesis) on zebrafish liver cells revealed that exposure to soluble Cd^2+^ (also released from CdTe QDs) significantly reduced NER repair capacity. This observation was attributed to Cd^2+^ interacting with DNA and hindering interactions, that lead to BPDE adduct formation, cellular uptake, intracellular distribution and possibly metabolism [19]. Indeed, Cd^2+^-inhibition of DNA repair of BPDE-induced DNA lesions has been studied previously [33,34]. Therefore, in addition to affecting the BER pathway, Cd has also been implicated in the interference of the NER pathway and suggests that the process of adduct formation is influenced by Cd^2+^ in the nucleus [35].

Apart from Cd ions, studies have shown that Fe ions can inhibit APE1’s enzymatic activity [36]. McNeill et al. demonstrated that Fe ions could prevent APE1 from excising a single, centrally located abasic site in a 26-mer oligonucleotide duplex [36]. To further assess the specificity of Fe dependent inhibition, and to better understand the effects in a physiological environment (that contains a whole array of different proteins), the study also investigated the impact of Fe ions on whole cell extracts. Similar results obtained on protein extracts provided substantiating evidence that Fe ions demonstrate specific inhibition of APE1 incision activity. Other studies have shown downregulation of APE1 in response to exposure by ENMs including AgNPs and TiO_2_ NPs [13,14]. Moreover, Jugan et al. has also shown impaired cellular DNA repair in lung epithelial cells in response to TiO_2_ NPs. The authors showed inhibition of both BER and NER pathway, as evaluated using a DNA repair microarray assay [37].

## 5. Conclusions & Future Considerations

The evolving development of ENMs has enabled innovative breakthroughs in a variety of sectors such as healthcare, manufacturing, agriculture and transportation [38,39]. The potential for human exposure to ENMs has increased what warrants a careful and thorough risk assessment of ENM interactions at the cellular level. Recent reports have shown that some ENMs may affect the fidelity of DNA repair processes/pathways. DNA damage occurs continuously as a result of aerobic respiration; thus, DNA repair pathways play an important role in maintaining genomic integrity. Potential perturbations in these pathways may result in biological alterations at the cellular/tissue/organism level.

Besides the adverse impact on genome integrity, it is worth mentioning that any defects in DNA repair genes involving aberrant regulation may impact a range of physiological functions and cellular processes. This is because the DNA repair pathway genes/proteins are also involved in apoptosis, transcriptional regulation, migration, telomere maintenance, chromatin remodelling and dNTP synthesis [7]. Altered DNA repair activity is further associated with tumour initiation and progression, and can result in increased resistance to DNA damage therapy.

Given the importance of DNA repair, it is vital to understand the role it plays in the genotoxic responses observed, and its specific impact on molecular interactions necessary for maintaining genomic stability. This review of the current literature demonstrates that there are several different factors that control DNA damage-induced mechanistic repair pathways. Important aspects to consider in the future, in order to more fully understand the potential impact of ENMs on DNA repair fidelity include, but are not limited to:

1. Characterisation of induced DNA damage lesions: a given ENM may have a primary mechanism for the induction of DNA damage, which triggers the initiation of a specific repair pathway. For example, metal and metal oxide based ENMs tend to cause oxidatively induced DNA damage, which is mainly repaired via the BER pathway. Therefore, characterising the type of DNA damage is critically important in future studies, as it will enable predictive models to be developed that can be used to predict which types of ENMs might affect specific DNA repair pathways.
2. Role of ions: inorganic NPs could via corrosion and dissolution release metal ions such as Cd^2+^, Fe^3+^, Zn^2+^, and Ag^+^ and hence influence the upregulation/downregulation (measured as excision activity) of various pathways. Additionally, metal ions released from ENMs have been shown to interact/bind with protein domains and amino acids of DNA repair proteins (e.g., zinc finger structures contained in the DNA repair protein, XPA) resulting in distorted protein structure and inefficient DNA repair activity [40]. Therefore, a thorough physicochemical characterisation of ENMs is imperative, to discriminate between the actual causative factor (ENM vs. metal ions), as the impact of ENMs on DNA repair pathway may be strongly associated with the presence of metal(s) either in their composition, or as undesirable impurities.
3. Dose-dependent DNA damage response: presently, the doses of ENMs administered in in vitro studies/test species to generate dose-response analysis may not mimic a potential human exposure level. This is because concentration-dependent activation of genes/pathways as well as transition in gene changes can be highly dose dependent. Therefore, dose ranges that are relevant to true exposure levels of ENMs need to be included when studying DNA damage responses pertaining to repair pathways. However, ENM exposure assessment currently presents a technical challenge and more work is needed to evaluate emissions of ENMs into the environment [41]. For example, it will be necessary to perform more thorough background measurements at workplaces to determine accurate occupational exposure levels, to develop appropriate metrics for ENM exposure assessments and to validate personal air samplers.
4. Method/technique: various techniques and methods with different endpoints are utilized for evaluating DNA damage repair and/or DNA damage responses, e.g., Western blots for translational changes/modifications and/or phosphorylation events; RT-PCR for transcriptional alterations; excision or incision assays for DNA repair enzyme activity; mass spectrometry methods for measuring adduct or lesion formation and multiplexed excision/synthesis assays for DNA repair enzyme inhibition activity [31]. Each method has its own sensitivity, specificity and endpoints, which makes it challenging to compare results across different studies. Hence, to enable an appropriate intra-laboratory/interlaboratory comparison of DNA damage repair responses, statistically appropriate analysis on normalised data must be performed in order to identify reproducible upregulation or downregulation of ENM-induced DNA repair responses.
5. Tissue specific detection /expression: different tissues and cell types (including primary cells, normal/cancer cell lines) exhibit varying DNA repair responses, which may correlate with the degree of DNA damage and susceptibility following exposure to some ENMs. Hence, it is imperative to measure the levels and activity of DNA repair genes and proteins, respectively, in all relevant cells, tissues or organs of interest as their expression and responses are largely “site-specific”.
6. Effect of acute vs. chronic exposure of ENMs: the type of exposure may affect the severity of the DNA damage and the resultant activation of specific DNA repair pathway(s). The human population may be exposed to natural, environmental or ENMs in a cumulative manner [42]. On the other hand, occupational, lifestyle or behaviour-related exposure to various nano-entities may induce acute responses [8,43]. Therefore, it is important to understand how various kinds of exposure scenarios dictate not only DNA damage, but also trigger specific repair pathways.
7. Effect of potential ENM artefacts on the interpretation of DNA damage repair or DNA damage response: as described in previous reports, the solution state physico-chemical properties of ENMs are not like the solution state physicochemical properties of chemicals [44]. Depending upon the category of ENMs under investigation, ENMs are prone to disparate rates of dissolution, aggregation/agglomeration phenomena, nutrient depletion and other behaviours that can potentially result in false-positive and/or false-negative responses in DNA damage repair and DNA damage response assays. These types of artefactual effects have been observed in many types of nanotoxicity [45] and nanoecotoxicity [44], but can be avoided by including appropriate experimental controls in the assays and having a thorough understanding of the assay variability parameters.

Although DNA repair process(es) are highly regulated and multifaceted involving cross-talk and overlapping functions, their evaluation in ENM-based studies contributes to an additional tier of complexity. Also, it is worth mentioning that it is extremely difficult at this stage to extrapolate from the in vitro/test species data discussed in this review, to actual ENMs exposure in vivo, e.g., outcomes on human health. The impact of ENM exposure on the fidelity of DNA repair processes is not fully understood at present and requires more in-depth investigation to elucidate the pathways of importance and the intrinsic or extrinsic ENM features that interfere with the maintenance of genomic stability.

## Figures and Tables

**Table 1:** Summary of studies showing ENMs-induced changes in components of DNA repair pathway. The arrow indicates upregulation or downregulation of the specified molecule at either the gene/protein level or enzymatic activity, depending on the analytical technique used.

Study	Analysis Technique Applied	Cell/Tissue Used	NP	DNA Repair Pathway and Its Corresponding Component Involved					
				Homologous Recombination Repair (HRR)	Non-Homologous End Joining (NHEJ)	DNA Damage Signalling (DDS)	Base Excision Repair (BER)	Nucleotide Excision Repair (NER)	Mismatch Repair (MMR)
AshaRani et al., 2012 [14]	mRNA and array hybridisation RT-PCR	Human lung fibroblast, IMR 90	AgNPs				↓ *APEX1*, *MUTYH* *MBD4* *OGG1*		
									↓ *PMS1* *MSH2*
				↓ *BRCA1*					
Kovvuru et al., 2014 [15]	DNA repair RT2 Profiler PCR array	Liver	AgNPs				↓ *APEX2* *NEIL3 NEIL1* *PARP1* *NTHL1* *MUTYH* *RPA1* *XRCC1*		
							↑ *TDG* *CCNO* *PARP2* *UNG*		
								↓ *RAD23B* *ERCC8* *XPC* *LIG1* *RAD23A* *RPA1*	
				↓ *RAD51/1* *RAD51*					
				↑ *RAD51C* *RAD52*					
Asare et al., 2015 [16]	PCR	Lung tissue	AgNPs	↑ *RAD51*					
						↑ *ATM*			
Satapathy et al., 2014 [17]	In Vivo Base Excision Repair (BER) Assay	Oral squamous cell carcinoma	QAgNPs				↓ LIG1 FEN1 POLB POLD1 POLE		
Van Berlo et al., 2010 [18]	mRNA expression	Lung tissue	Carbon				↑ *OGG1* *APEX1*		
Tang et al., 2013 [19]	RT-PCR	Daphnia pulex	CdSO4 or CdTeQDs		↑ *Ku80*				
							↑ *OGG1*		
								↑ *XPC* *XPA*	
Tang et al., 2015 [20]	RT-PCR	Daphnia pulex	CdTe/ZnS				↑ *OGG1*		
								↑ *XPA* *XPC*	
Ahamed et al., 2010 [21]	Western blotting	Human pulmonary epithelial cells (A549)	CuO	↑ RAD51					
									↑ MSH2
Khatri et al., 2013 [22]	RT-PCR	THP-1, Primary human nasal, Small airway epithelial	ENMs emitted from photocopiers	↑ *RAD51*					
					↑ *Ku70*				
Prasad et al., 2013 [13]	Western blot (phosphorylation)	Human dermal fibroblasts	TiO2			↑ Activation of ATM/Chk2 DNA damage signalling pathway			
El-said et al., 2014 [23]	RT-PCR	HepG2	TiO2				↑ *APEX1* *MBD4*		
						↑ *ATM*			
Hanot-Roy et al., 2014 [24]	Western blot (phosphorylation)	Alveolar macrophages (THP-1), Epithelial cells (A549), Human Pulmonary Endothelial Cells (HPMEC-ST1.6R cells)	TiO2			↑ ATM ATR			
Pati et al., 2016 [25]	Western blot	Macrophages	Zinc oxide nanoparticles (ZnO-NPs)				↓ POLB FEN1		

**Table 2:** Function of important enzymes/proteins involved in the major DNA repair pathways.

Enzyme/ Protein	Function
**DDS Pathway**	
ATM (ataxia-telangiectasia mutated)	Cell cycle checkpoint kinase protein, which belongs to the PI3/PI4- kinase family. Serves as a DNA damage sensor and regulator of a wide variety of downstream proteins, including, 1) Tumour suppressor protein p53 and 2) Serine/threonine protein kinase that activates checkpoint signalling upon double strand breaks (DSBs), apoptosis, and genotoxic stresses.
ATR Rad3-related kinase	PI3 kinase-related kinase family member (like ATM), which phosphorylates multiple substrates on serine/ threonine residues (that are followed by a glutamine) in response to DNA damage or replication blocks. Causes cell cycle delay, in part, by phosphorylating checkpoint kinase (CHK)1, CHK2, and p53.
CHK1 and CHK2 (Checkpoint kinase 1 and 2)	Downstream protein kinases of ATM/ATR, which play an important role in DNA damage checkpoint control.
**BER Pathway**	
APEX1 (Apurinic/apyrimidinic endonuclease 1)	Multifunctional DNA repair enzyme, apurinic/apyrimidinic endonuclease 1/redox factor-1 (APE1/Ref-1) responsible for abasic site cleavage activity. Plays a critical role in the DNA base excision repair (BER) pathway and in the redox regulation of transcriptional factors. Activated/ induced by oxidative DNA damage. Localisation signals, post-translational modifications and dynamic regulation determines the localisation of APE protein in the nucleus with subcellular localization in the mitochondria, endoplasmic reticulum and cytoplasm.
APEX2 (Apurinic/apyrimidinic endonuclease 2)	AP endonuclease 2 is characterized by a weak AP endonuclease activity, 3′-phosphodiesterase activity and 3′- to 5′-exonuclease activity. Involved in removal of mismatched 3′-nucleotides from DNA and ATR-Chk1 checkpoint signalling in response to oxidative stress.
(POLB) DNA polymerase β	Contributes to DNA synthesis and deoxyribose-phosphate removal.
(FEN1) Flap endonuclease 1	Possesses 5′–3′ exonuclease activity and cleaves 5′ overhanging “flap structures” in DNA replication and repair.
LIG1 (Ligase 1)	Seals SSB ends.
MBD4 (methyl-CpG binding domain protein 4)	Belongs to a family of nuclear proteins that possess a methyl-CpG binding domain (MBD). These proteins bind specifically to methylated DNA, possess DNA N-glycosylase activity and can remove uracil or 5-fluorouracil in G:U mismatches.
MUTYH (mutY DNA glycosylase)	Serves as DNA glycosylase (excises adenine mispaired with 8-oxoguanine). Maintains chromosome stability by inducing ATR-mediated checkpoint activation, cell cycle arrest and apoptosis.
NEIL1, NEIL3 (Nei-like 1; Nei-like 3)	Generate apurinic/apyrimidinic (AP) sites and/or SSBs with blocked ends.
NTHL1	Serve as oxidized base-specific DNA glycosylases that remove oxidized and/or mismatched DNA bases.
OGG1 (8-oxoguanine DNA glycosylase)	Excises and repairs oxidatively damaged guanine bases in DNA, which occur as a result of exposure to ROS.
PCNA (Proliferating cell nuclear antigen)	Co-factor for DNA polymerase and essential for DNA synthesis and repair.
PARP1 (Poly ADP ribose polymerase)	PARP1—serves as sensor of SSBs.
XRCC1 (X-ray repair cross-complementing protein 1)	XRCC1—serves as a scaffold for recruiting and activating BER proteins.
**NER Pathway**	
RPA1 (replication protein A1)	Largest subunit of the replication protein A (RPA), the heterotrimeric single-stranded DNA-binding protein involved in replication, repair, recombination and DNA damage check point activation.
XPC (xeroderma pigmentosum group C protein)	Recognizes bulky DNA adducts. Pairs up with RAD23 and helps in the assembly of the other core proteins involved in NER pathway progression.
XPA (xeroderma pigmentosum group A protein)	Attaches to damaged DNA, interacts along with other proteins in the NER pathway to unwind, excise and replace the damaged DNA.
**HRR Pathway**	
BRCA1/ BRCA2 (breast cancer type 1 and type 2 susceptibility proteins)	BRCA1 and BRCA2 are coded by human tumour suppressor genes that are involved in DNA damage repair, cell cycle progression, transcription, ubiquitination and apoptosis. Aberrant proteins coded by mutated genes are found in hereditary breast and ovarian cancers; activation of various kinases in response to DNA-damage have been shown to phosphorylate sites on BRC1 and BRC2 in a cell cycle-dependent manner.
RAD51	Involved in the homologous recombination and repair of double strand DNA breaks.
**NHEJ Pathway**	
Ku	Ku, a heterodimer of two related proteins, Ku70 and Ku80, is involved in DSB repair and V(D)J recombination.
LIG4 (Ligase 4)	LIG4 is the DNA ligase required for, and specific to, c-NHEJ. It catalyzes the same ATP-dependent transfer of phosphate bonds that results in strand ligation in all eukaryotic DNA repair. LIG4 is the only ligase with the mechanistic flexibility to ligate one strand independently of another or to ligate incompatible DSB ends as well as gaps of several nucleotides.
XRCC4 (X-ray repair cross-complementing protein 1)	XRCC4 is a non-enzymatic protein that is required for the conformational stability and functioning levels of LIG4. XRCC4 interacts with LIG4 facilitated by carboxy-terminal repeats at the LIG4 carboxyl terminus, resulting in a coiled-coil like conformation. Most of the enzymatic domain of LIG binds to and interacts with XRCC4, except for the small region implicated in DNA binding.
